# The silent spread of Hepatitis E in India - from epidemiological insight to public health action: a comprehensive review

**DOI:** 10.1007/s15010-025-02661-2

**Published:** 2025-10-16

**Authors:** Snigdha Maity, Shivam Chowdhary, Akila Swaminathan, Nidhi Ashtaputre, Piya Paul Mudgal, Chiranjay Mukhopadhyay

**Affiliations:** Manipal Institute of Virology, Manipal Academy of Higher Education, Manipal, India

**Keywords:** Hepatitis E virus, Waterborne epidemics, Zoonotic transmission, Liver disease, Maternal health, Sanitation

## Abstract

Hepatitis E represents an increasingly significant yet often overlooked public health issue in India, contributing substantially to both sporadic hepatitis cases and widespread waterborne outbreaks. Hepatitis E virus (HEV) is the foremost cause of acute viral hepatitis (AVH) in India and spreads primarily through contaminated water. Genotype-specific differences in transmission routes, ranging from enteric routes in developing regions to zoonotic routes in industrialized settings, underscore the complexity of its epidemiology. Vulnerable populations such as pregnant women, animal handlers, and immunocompromised individuals face a markedly increased risk of severe disease outcomes, including fulminant hepatic failure and chronic infection, in cases of coinfection with hepatitis B virus.

This comprehensive review delves into Indian epidemiological trends, clinical features, diagnostic approaches, and current management options for HEV. While most infections are self-limiting, ribavirin has shown efficacy in select high-risk populations. However, the absence of an approved vaccine in India remains a critical gap in preventive strategies. Emerging therapeutics and vaccine candidates are currently in various stages of development. However, challenges such as the genetic diversity of HEVs, lack of long-term efficacy data, and limited public awareness hinder progress.

This review emphasizes the urgent need for strengthened national surveillance systems, improved water and sanitation infrastructure, and integrated public health policies tailored for high-risk groups. A multipronged approach that combines epidemiological vigilance, clinical preparedness, and policy-driven interventions is imperative to halt the silent transmission of hepatitis E in India.

## Introduction

Hepatitis E virus (HEV) has been increasingly identified as a zoonotic pathogen of concern and is considered the leading cause of AVH, a condition characterized by hepatic inflammation and damage, typically resolving within 4 to 6 weeks [1]. Although self-limiting, HEV can cause chronic hepatitis, fibrosis, cirrhosis, and may contribute to the development of hepatocellular carcinoma in immunocompromised individuals, impacting morbidity. It disrupts liver function, causing jaundice and, rarely, fulminant hepatitis, leading to liver failure. HEV accounts for an estimated 20 million annual infections globally, underscoring its significant public health burden [2, 3]. Notably, HEV infection contributes to substantial mortality and morbidity worldwide, especially among pregnant women, who face a heightened risk of severe complications [4]. The evolving spectrum of clinical presentations and epidemiology highlights the serious global health impact of HEV, necessitating further research into its pathophysiology [5].

## Hepatitis E virome

**Fig. 1:**
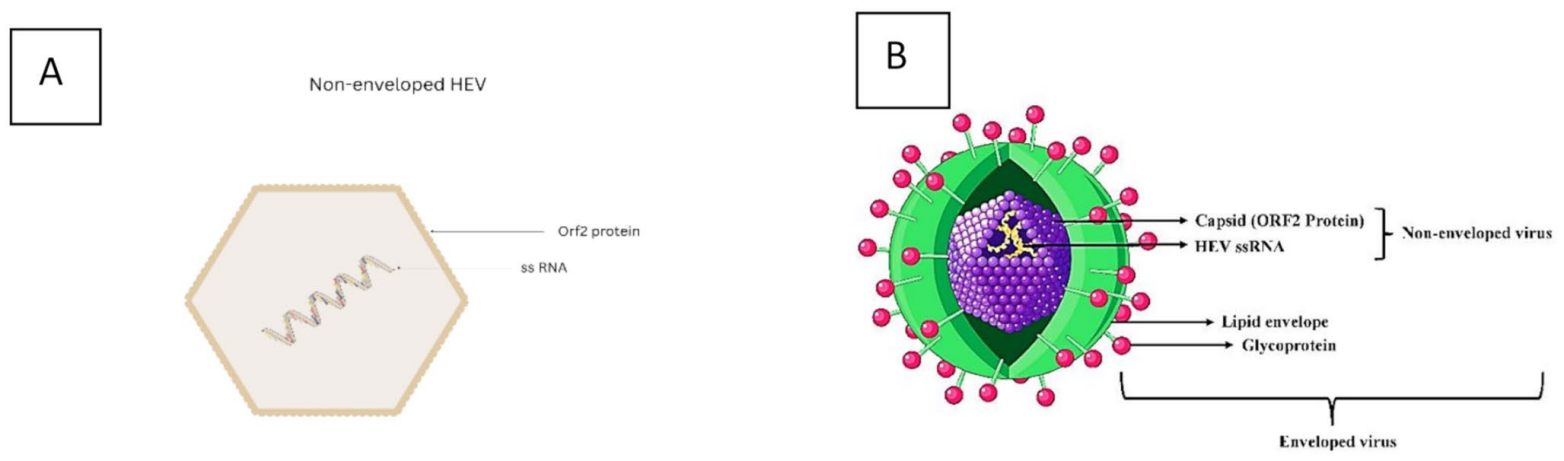
The dual structure of HEV. HEV exists in two forms depending on its environment. A. Non-enveloped HEV consists of a naked capsid protein (ORF2) that encloses ssRNA genome. These virions are shed in the feces, which are stable outside the body and optimized for fecal-oral transmission. B. Enveloped HEV, also known as quasi-enveloped HEV, is formed in the bloodstream and infected hepatocytes of the host. It consists of capsid protein and ssRNA genome surrounded by host-derived lipid envelope. This envelope masks viral antigens, facilitating immune evasion and persistence within the host.Adapted from Servier (10/8/2024). Enveloped Virus. Smart Servier Medical Art Source. Available from https://smart.servier.com/smart_image/smart-hepatitis-virus/

HEV is a non-enveloped virus with an icosahedral capsid (27–34 nm) carrying single-stranded, positive-sense RNA (Figure 1). The *Hepeviridae* family, *Orthohepevirus* genus [3], was initially recognized as non-A, non-B hepatitis. HEV is transmitted primarily through the enteric route in countries with poor sanitation and unhygienic conditions [4]. Since HEV is excreted in feces, it is capable of surviving outside the host for long periods. In blood and culture supernatants, HEV virions are quasi-enveloped by acquiring a host-derived lipid membrane via the exosomal pathway. When the virus buds into multivesicular bodies, the ORF3 protein assists its release by exocytosis. The envelope assists the virus in evading neutralizing antibodies and escaping the host’s immune system. The envelope is lost as the virions pass through the biliary tract. Hence, in the feces, non-enveloped viral particles are shed and are infectious [6]. Furthermore, the emergence of zoonotic transmission, with genotypes 3 and 4, expands the transmission pathways beyond traditional enteric routes, adding complexity to its epidemiology.

## Tracing the spread: the epidemiology of hepatitis E

### Global pathways of HEVs

HEV represents a considerable global health challenge, with annual estimates indicating 2 million infections, 3.3 million symptomatic cases, and over 70,000 fatalities [7, 8]. Approximately 33% of the global population, exceeding 2 billion individuals, resides in HEV-endemic regions [9]. The virus was first identified during a significant outbreak in Kashmir Valley, India, in 1978, causing 52,000 cases and 17,000 fatalities [10]. HEV is endemic across Asia, the Middle East, Central America, and Africa, with India demonstrating high endemicity [1]. In India, Genotype 1 (GT1) HEV, which primarily infects humans, dominates epidemics and sporadic outbreaks [1, 11]. While GT3 and GT4 HEVs circulate among wild mammals, animal-to-human transmission is rare [12]. Globally, GT3 is common in Europe; GT4 is common in China, Korea, and Japan; and GT2 is common in West Africa and Mexico, whereas GT1 is widespread in Asia and Africa [13, 14]. Zoonotic transmission, primarily via porcine reservoirs and consumption of undercooked pork, is common in developed countries [1]. Initially, HEV transmission in developing nations occurred through fecal-contaminated water [15]. Despite improved sanitation, HEV remains prevalent in resource-limited regions lacking clean water. Seroprevalence data on anti-HEV IgG (immunoglobulin G) indicate that approximately 12.47% of the global population has experienced HEV in the past, with Asia (21.76%) and Africa (15.80%) exhibiting the highest rates. Globally, an estimated 110 million individuals have anti-HEV IgM (Immunoglobulin M) and 15 million have detectable HEV RNA [9]. However, underdiagnosis and underreporting in developing countries suggest that the true burden is significantly greater than current estimates [16]. Other HEV GT5 and GT6 have been isolated from wild boars in Japan, but no human infections have been documented. However, the virus can be transmitted to *Cynomolgus macaques* in laboratory settings, which indicates a potential risk for the transmission from animals to humans. At least one immunocompromised person developed chronic hepatitis E from GT7 after ingesting camel milk and meat in the Middle East, where dromedary camels were found infected. GT8, which is found in Bactrian camels in China, can infect monkeys but not typically humans [17–19].

### Mapping HEV: prevalence across the Indian subcontinent

Hepatitis E, a significant cause of AVH in India, is characterized by numerous regional outbreaks. The 1991 Kanpur epidemic, which affected more than 79,000 people, was among the largest, with higher attack rates in adult males [20]. Subsequently, between 2011 and 2013, India’s Integrated Disease Surveillance Programme (IDSP) documented 78 HEV outbreaks and 22,671 cases with 152 deaths from 2013 to 2018 [21]. An HEV genotype 1a outbreak in Raipur (2014), linked to sewage-contaminated water, recorded a 68.8% attack rate, resulting in 23 deaths, including 8 pregnant women [22]. The first major HEV epidemic occurred in Delhi during 1955–1956, and subsequently, retrospective investigations identified the etiological agent as HEV. The outbreak involved more than 29,000 cases of jaundice due to an overflow of sewage-contaminated water from the Yamuna River during monsoon rains. Approximately 266 deaths were recorded with a higher prevalence among pregnant women. The 1978–1979 Kashmir epidemic (November to April) occurred in over 200 villages of the Gulmarg area and affected an estimated population of 600,000. The outbreak resulted in 20,083 cases due to contaminated water supplies. The high mortality rates (20 –30%) among pregnant women supported that HEV is a distinct disease. Subsequently, from 2015 to 2017, Kashmir reported 10 outbreaks with 393 cases [13, 14]. These outbreaks consistently involve fecal contamination of drinking water, confirmed by anti-HEV IgM positivity [10], highlighting waterborne transmission in India. However, our understanding of HEV transmission has evolved. A study in the Amritsar region revealed that HEV was the leading cause of waterborne hepatitis, peaking in the 21–30 years age group [16]. Furthermore, socioeconomic factors, particularly poverty and poor sanitation, are correlated with increased HEV incidence. Vertical transmission to newborns has been reported, although it is self-limiting. Seroprevalence studies among blood donors and patients have revealed widespread anti-HEV IgG and IgM positivity, indicating past, active, and subclinical infections. Coinfection with HBV has also been reported [23–26].

## HEV’s evolving landscape: 2015–2024

HEV is a globally recognized enteric pathogen, with diverse transmission routes, including zoonotic, parenteral, and vertical [5]. A systematic review (1987–2023) of Southeast Asia revealed increasing anti-HEV IgG and IgM prevalence. The pooled IgG prevalence was 21.03%, with the highest in Myanmar (33.46%) and the lowest in Malaysia (5.93%). IgM was highest in Indonesia (12.43%) and lowest in Malaysia (0.91%), underscoring the need for targeted public health interventions [7].

Global Burden of Disease data (1990–2019) show a global decline in HEV incidence; however, high-incidence regions such as sub-Saharan Africa, Oceania, South Asia, and Central Asia bear the greatest burden [7]. A 2024 outbreak in Chad, with more than 2000 suspected cases and 0.3% case fatality, demonstrated persistent vulnerability among displaced populations, highlighting sanitation and hygiene needs [8]. Disparities in healthcare access and sanitation remain prevalent, particularly in low- and middle-income countries. Concurrently, affluent countries, notably Europe, have seen rising native cases linked to GT3 from undercooked pork [9].

The evolution of hepatitis E from an overlooked tropical disease to a global concern is marked by an increase in chronic infections, particularly among immunocompromised patients [1]. The WHO emphasizes vaccination and better sanitation to curb HEV, but broader efforts are needed. A meta-analysis revealed that country, testing method, and age distribution influence HEV IgG incidence in nonendemic regions [12]. Despite a highly efficacious recombinant vaccine, Hecolin, being approved in 2011 for use in China, global vaccination remains largely unimplemented [11]. Effective combat requires specialized public health approaches, enhanced diagnostics, and equitable healthcare access, as the true burden is underestimated [5].

HEV causes 30–70% of acute sporadic hepatitis cases and large outbreaks, primarily in low- and middle-income countries. Outbreaks involving HEV types 1 and 2 are linked to inadequate sanitation, fecal contaminated water, population displacement, low socioeconomic conditions, and natural disasters such as floods. In contrast, rare sporadic cases in developed nations are mainly due to zoonotic HEV genotype 3, acquired from the consumption of undercooked meat, particularly pork [3, 13, 14].

## Urban vs. rural: how environments shape transmission

**Fig. 2:**
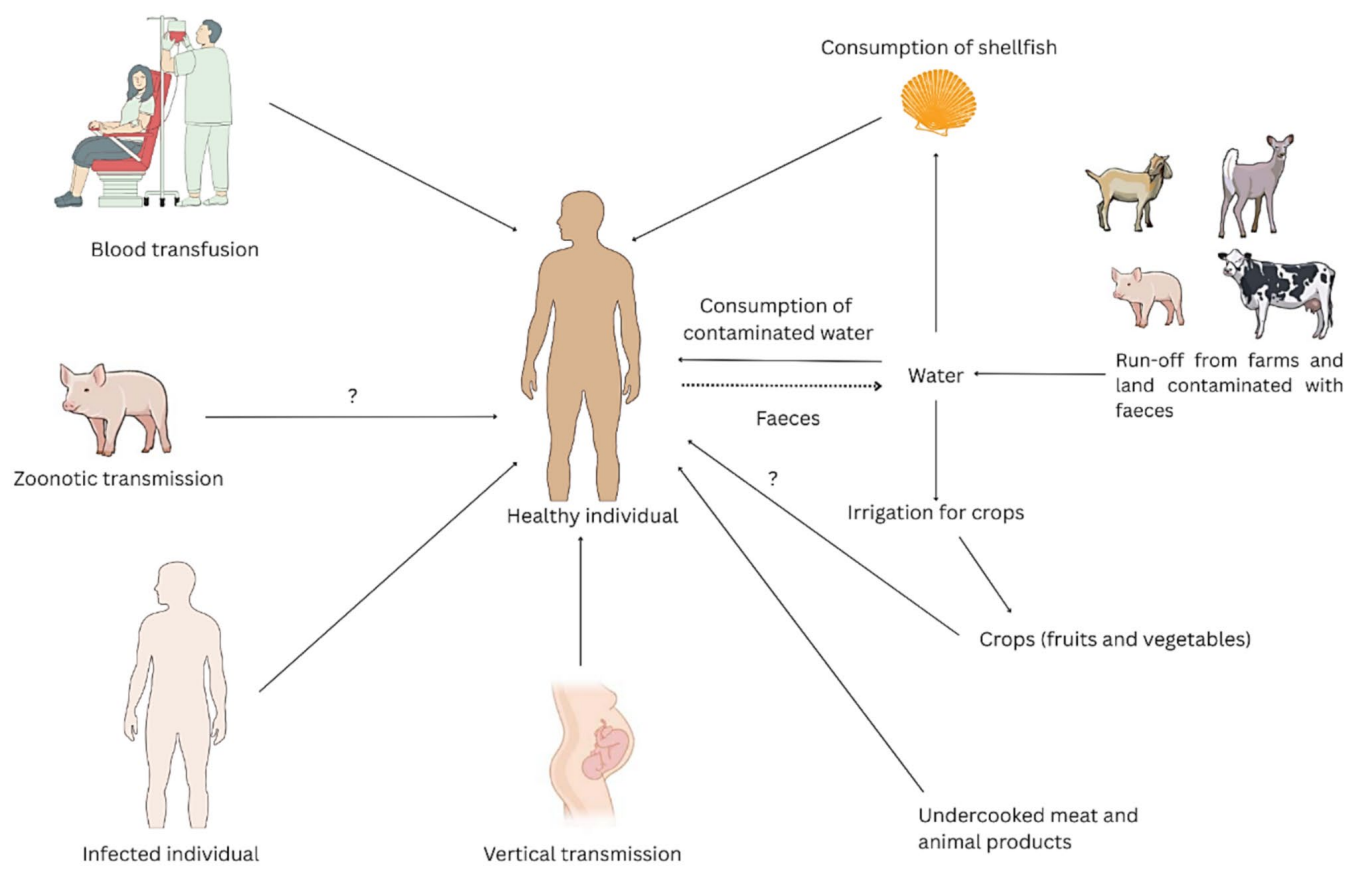
Transmission routes of HEV. HEV is transmitted via contaminated water, feces, and shellfish; zoonotic transmission is also seen after direct contact with animals and consumption of undercooked meat. Although less frequent, HEV is also transmitted vertically and through blood transfusions [28]. Diagram created in Canva. Icons and illustrations sourced from Canva’s content library and adapted from NIAID Visual & Medical Arts. (8/10/2024)

HEV transmission, primarily via the fecal‒oral route, is influenced by water quality, sanitation, hygiene, and food practices (Figure 2) and varies between rural and urban settings. Rural areas often face greater challenges [12], as floods, contaminated water sources, inadequate sanitation, and poor hygiene increase infection risk [13, 14]. Additionally, agricultural practices involving close contact and handling of infected livestock, especially pigs, can contribute to zoonotic transmission in rural populations [15]. A lack of awareness, poor socioeconomic status, and limited healthcare access in rural areas delay diagnosis and treatment [16].

High population density and rapid urbanization in cities favour HEV spread, as overcrowding and poor sanitation accelerate transmission [13, 14, 20]. Migration to urban areas and informal settlements further elevates risk. Despite better infrastructure, transmission continues due to poor hygiene, contaminated water, and inadequate sewage [27]. Although less frequent, zoonotic transmission from shellfish and contaminated animal products remains a risk [15]. Extreme weather, such as droughts and floods, can worsen water scarcity, leading to compromised hygiene and increasing HEV transmission [12].

## The dual faces of HEVs: endemicity explained

Hepatitis E virus presents two distinct epidemiological patterns on the basis of its four genotypes (Table 1). In developing countries, genotypes 1 and 2 are widely prevalent; they spread via the fecal‒oral route and cause acute outbreaks, mainly in young adults and pregnant women, whereas chronic cases are rare [29]. In contrast, genotypes 3 and 4 in developed countries cause sporadic zoonotic infections, which sometimes progress to chronic disease in older and immunocompromised individuals [13–16].

**Table 1:** Two distinct epidemiological patterns of hepatitis E

CHARACTERISTIC	HIGH ENDEMICITY	LOW ENDEMICITY
Geographical location	*Developing countries- South/Central Asia*,* Africa*,* Central America*	*Developed countries-* *North and South America*,* Europe*,* Australia*,* East Asia*,* South Africa*
HEV Genotypes	*1 and 2*	*3 and 4*
Transmission Route	*Fecal-oral*,* contaminated water*	*Zoonotic*,* blood transfusion*
Affected Population	*Young adults*,* pregnant women*	*Elderly*,* immunocompromised*
Chronic Infection	*Rare*	*Common in immunocompromised*

## Hepatitis E: recognizing the signs & complications

### Acute hepatitis E

HEV infection is often mild or asymptomatic, with a 2–10-week incubation period [29]. Symptomatic cases begin with a prodromal phase marked by low-grade fever, fatigue, nausea, vomiting, and anorexia, followed by signs of acute hepatitis, including dark urine, pale stools, myalgia, abdominal pain, itching, hepatomegaly, and jaundice [4]. The levels of liver transaminases, bilirubin, alkaline phosphatase, and γ-glutamyl transferase are often elevated. Although alanine aminotransferase (ALT) levels in typical HEV patients can reach ~ 1500 IU/L, some patients may present minimal or normal levels despite active viremia. Jaundice occurs in approximately 40% of acute cases. In immunocompetent individuals, infection usually resolves spontaneously with rest and supportive care within weeks [30, 31].

Severe complications such as acute liver failure (ALF) or fulminant hepatitis can rarely occur, especially in pregnant women and those with preexisting liver conditions. ALF involves rapid loss of liver function within eight weeks of symptom onset and is characterized by coagulopathy (International Normalized Ratio ≥ 1.5) and encephalopathy without preexisting cirrhosis [32]. ALF is characterized by jaundice, encephalopathy, severe liver injury, bleeding, and increased risk of death, necessitating close monitoring and, in some cases, emergency liver transplantation [32].

### Chronic hepatitis E

Chronic Hepatitis E, characterized by symptoms persisting beyond 6 months, primarily affects immunocompromised individuals such as transplant recipients, patients on immunosuppressants, and those with pre-existing liver disease. HIV–HEV coinfected individuals, pregnant women, and cancer patients on chemotherapy are also at increased risk for severe complications and extrahepatic manifestations [8, 9]. Clinical features include elevated transaminase levels, progressive liver damage, cirrhosis, and possible mortality [33]. Extrahepatic complications include pancreatitis; neurological disorders such as Guillain–Barré syndrome; myelitis; encephalitis; neural amyotrophy; hematological abnormalities; renal injury [33]; and cryoglobulinemia and rheumatologic manifestations [27]. The diagnosis of chronic HEV requires a persistently positive reverse transcriptase polymerase chain reaction test for HEV RNA in serum or stool samples obtained at least three to six months after the initial diagnosis [9].

### Extrahepatic

**Fig. 3:**
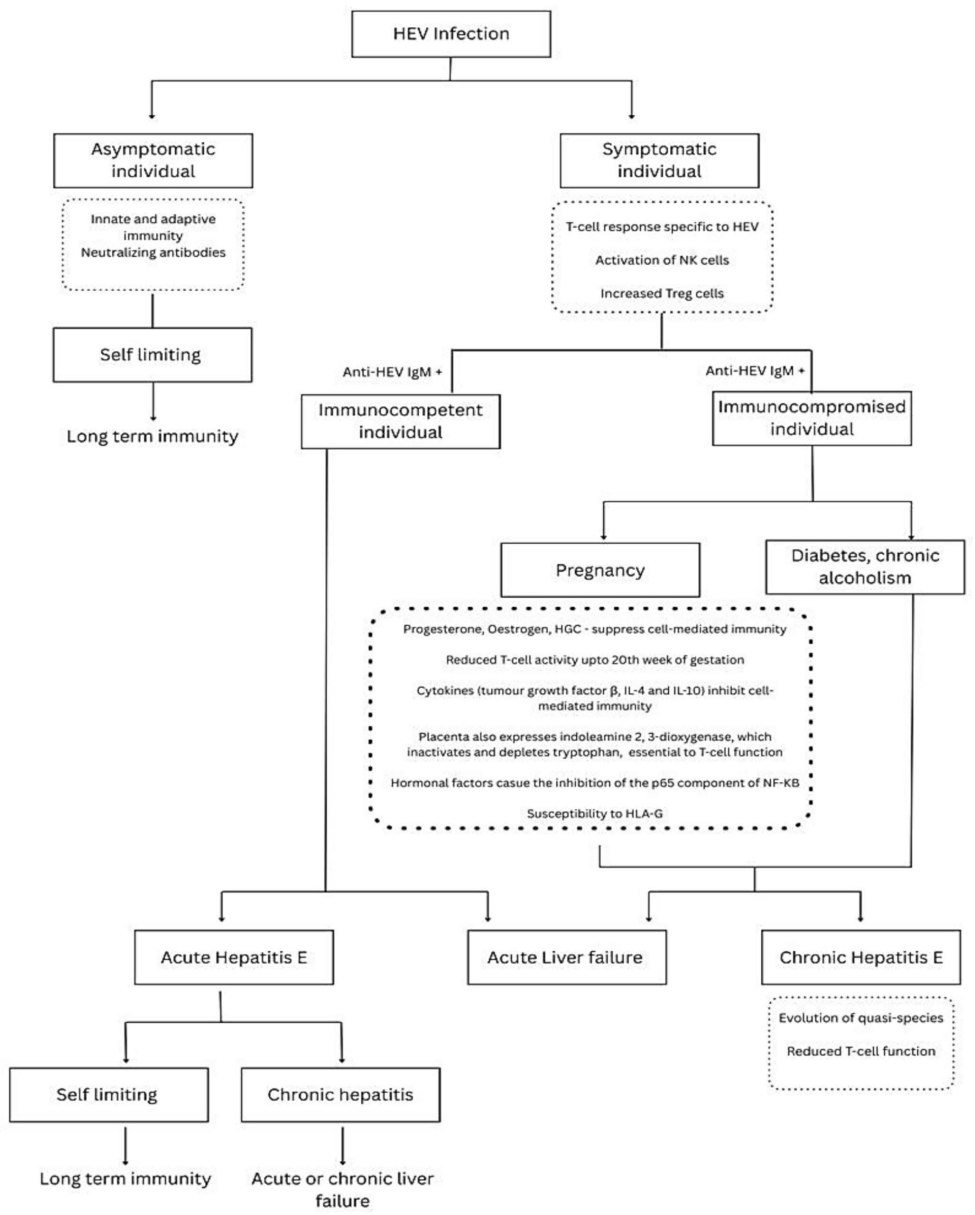
Clinical manifestations and immune response in HEV infection. Created via Canva elements

These include neurological, renal, and rheumatological complications. The neurological issues most commonly include pyramidal signs, ataxia, myopathy, encephalitis, cognitive impairment, and various neuropathies. Renal complications, including glomerulonephritis, IgA nephropathy relapses, and nephrotic syndrome, have been reported, especially in transplant recipients. Rheumatological manifestations, including arthralgia, myalgia, dermatological rashes, and cryoglobulinemia, are primarily associated with chronic infection [33]. Figure 3 illustrates the clinical features and immune response of HEV.

## HEV in pregnancy: a critical concern

**Fig. 4:**
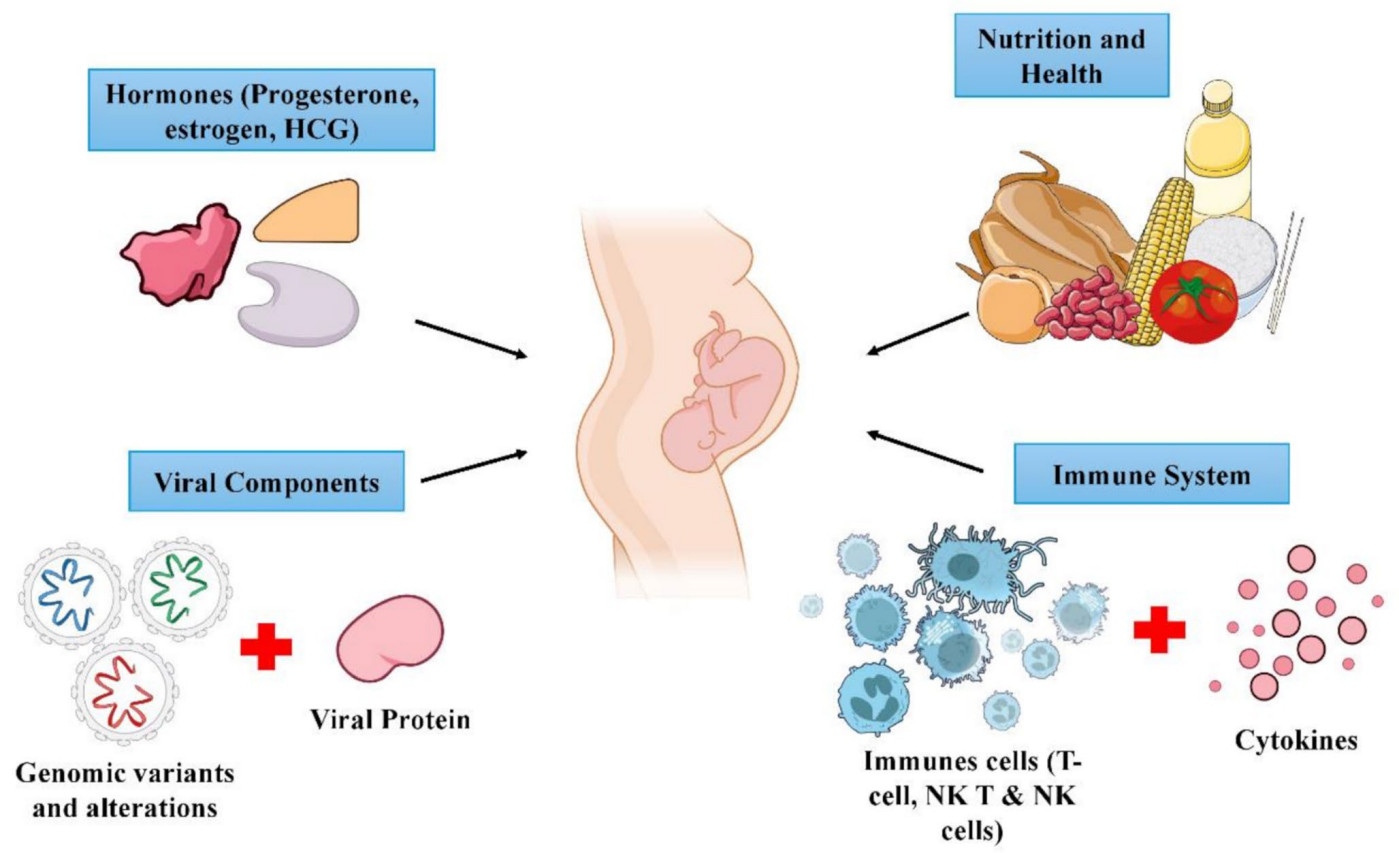
Mechanisms underlying HEV-induced severe liver injury in pregnancy [24, 34]. Image adapted from Servier Medical Art (https://smart.servier.com/), licensed under CC BY 4.0 (https://creativecommons.org/licenses/by/4.0/) and NIAID Visual & Medical Arts. (10.8.2024)

HEV infection during gestation, especially in the third trimester, is linked to high maternal and fetal mortality, with case fatality rates reaching 20–25% [33]. GT1 infections are associated with greater disease severity, including complications such as ALF and fulminant hepatitis, especially in the second and third trimesters. Vertical transmission may lead to adverse outcomes such as miscarriage, stillbirth, premature delivery, postpartum haemorrhage, and both neonatal and maternal mortality [24]. The underlying mechanisms of severe liver injury in pregnancy remain unclear, but hormonal and immunological alterations, combined with HEV genome variability, are implicated [1] (Figure 4). In contrast, breastfeeding is generally considered safe in the absence of active symptomatic infection [34].

## Illuminating insights (via biomarkers)

To assist in diagnosing hepatitis E and understanding its progression, various biomarkers have been identified, including those in plasma, urine, and microRNA (miRNA) profiles, reflecting the host’s response to HEV.

### Plasma and urine biomarkers

Taneja et al. identified plasma transthyretin and urinary α1-microglobulin as potential biomarkers for acute hepatitis E. Preliminary data also suggest that urinary zinc alpha glycoprotein levels are lower in fulminant patients than in acute HEV patients, but these findings require further validation. Five potential peptide peaks were further identified through mass spectrometry, indicating strong diagnostic potential for differentiating hepatitis E patients from healthy controls [35].

### MicroRNA signature biomarkers

Costafreda et al. reported that certain miRNA signatures, including the upregulation of miR-122 and miR-194 in acute hepatitis E patients and the downregulation of miR-125b-5p and miR-192-5p in chronic hepatitis E patients, could serve as biomarkers [36]. While these biomarkers show promise for improving hepatitis E diagnosis, the variability in HEV outcomes necessitates further research to validate findings and identify additional markers.

### Optimizing biomarker profiles for sporadic HEVs

A study of 271 sporadic acute hepatitis cases revealed 91 confirmed HEV infections on the basis of RNA, IgM, or significant increases in IgG. It revealed frequent false negatives with individual markers, emphasizing the need for a combined diagnostic approach using HEV RNA, IgM, and rising IgG, especially in regions with variable endemicity and reinfection rates [37].

### Interleukin-18 (IL-18) as an indicator of HEV exposure and transfusion risk

A cross-sectional study of Mexican blood donors revealed 9.4% HEV seropositivity and detected HEV RNA in one pooled seropositive sample, highlighting the risk of transfusion-related transmission. Elevated IL-18 and interferon gamma (IFN-γ) levels are associated with HEV seropositivity, suggesting that IL-18 is a potential biomarker of HEV exposure, which is consistent with profiles observed in acute cases [38].

## HEV: a challenging diagnosis

Early HEV diagnostic methods, such as electron microscopy, fluorescent antibody assays, and antigen detection, are labor-intensive and complex, limiting their clinical use. Cloning of the HEV genome later enabled significant advances in molecular and serological diagnostics [35]. The European Association for the Study of the Liver guidelines recommend including HEVs in the differential diagnosis for viral hepatitis and drug-induced liver injury, especially during chronic liver disease flares or in immunocompromised patients and transfusion recipients with abnormal LFTs (liver function tests) [39].

Clinically, HEV is indistinguishable from other AVHs requiring serological confirmation. Diagnosis is typically considered during outbreaks, suspected water contamination, severe hepatitis during pregnancy, or when hepatitis A is excluded. Serum anti-HEV IgM confirms recent infection and lasts 3–4 months, sometimes up to a year [40]. Anti-HEV IgG antibodies, while long-lasting, suggest past exposure, with variable persistence [41]. However, a diminished antibody response in immunocompromised patients makes serology unreliable [40].

Molecular testing, such as HEV RNA detection in conjunction with IgM antibody detection, is crucial for confirming an acute or chronic active infection. RNA appears ~ 3 weeks post-exposure, with fecal shedding lasting 4–6 weeks [36, 41]. In acute HEV, the serum bilirubin level typically exceeds 2.5 mg/dL, and ALT level increases over tenfold (29–33 U/L in men, 19–25 U/L in women) [42]. The diagnosis of HEV in immunocompetent individuals should combine serology and molecular methods, while PCR is critical for immunocompromised patients and for identifying chronic HEV, defined by HEV RNA persistence for over 3 months [13, 14]. In low-resource settings, epidemiological evidence may support diagnosis [13]. The World Health Organization (WHO) has set nucleic acid amplification test-based standards for blood and plasma safety [43]. The Indian Council of Medical Research-National Institute of Virology has developed enzyme-linked immunosorbent assays (ELISAs), PCR assays, and rapid strip tests for waterborne HEVs to aid diagnosis in resource-limited areas [44].

## Point-of-care diagnostics: bringing the lab to the patient

Currently, no Food and Drug Administration (FDA)-approved point-of-care test (POC) exists for HEV [37]. However, significant progress has been made with CRISPR-Cas13a (clustered regularly interspaced short palindromic repeats - CRISPR-associated proteins) systems, lateral flow assays, and molecular POC tests, which offer high sensitivity, specificity, and rapid results and are especially valuable in resource-limited settings [45, 46]. Ying et al. developed a fluorescent microbead-based immunoassay (FMIA) for rapid point-of-care HEV antigen detection, which demonstrated high sensitivity (92%) and specificity (100%). This FMIA correlated well with HEV RNA levels and commercial assays, proving valuable for the rapid and simple diagnosis of acute HEV in developing and rural settings [47]. Li et al. developed a rapid HEV RNA diagnostic method using reverse-transcription recombinase polymerase amplification (RT-RPA) combined with a lateral flow strip. The assay showed high sensitivity (detection limit: 1.0 × 10² copies/mL) and specificity, with no cross-reactivity to other hepatitis viruses, and effectively detected HEV GTs 3 and 4 [48, 49]. CRISPR-Cas13a-based systems have shown great promise for HEV RNA detection, with high sensitivity and specificity. Detection limits reach 12.5 IU/mL for fluorescence assays and 200 IU/mL for strip formats, identifying HEV genotypes 1–4 without cross-reactivity [50]. Advanced lateral flow assays, such as reverse transcription recombinase-aided amplification with lateral flow dipstick (RT-RAA-LFD) and quantitative real-time reverse transcription recombinase-aided amplification (qRT-RAA), enable rapid HEV RNA detection with high sensitivity and specificity. RT-RAA-LFD is complete in 15 min at 39 °C (limit of detection (LOD): 247 copies/µL), whereas qRT-RAA is completed in 20 min at 42 °C (LOD: 25 copies/µL), making it a valuable tool for rapid screening, including in veterinary settings [51].

## Treatment roadmaps

Effective management of hepatitis E requires a tailored approach on the basis of disease severity, patient risk factors, and immune status. While most acute cases are self-limiting and require supportive care, specific antiviral therapies are considered for chronic infections and high-risk groups. Table 2 summarizes current and emerging HEV treatment strategies.

**Table 2:** Summary of current therapeutic interventions for HEV

Treatment Category	Approach/Drug	Mechanism of Action/Use	Clinical Notes/Limitations	Reference
Supportive care (Acute Hepatitis E)	Ursodeoxycholic acid & Cholestyramine	*Symptomatic management-* used to manage symptoms like jaundice, pruritus, and fatigue.	Most acute HEV cases are self-limiting; no antiviral therapy is needed, monitor LFTs and bilirubin for progression.	[52]
Antiviral Therapy (Chronic Hepatitis E)	Ribavirin	Inhibits viral RNA synthesis. Normalizes liver enzymes and reduces viral load in chronic cases.	First-line therapy (approx. 3 months) with optimal dose of 1.8–2.3 mg/L for Solid Organ Transplant (SOT) recipients; contraindicated in pregnancy due to teratogenicity; EASL advises use only if immunosuppression reduction fails.	[53]
	Pegylated Interferon -α	An immunomodulator used in liver transplant or HIV patients with chronic HEV.	Risk of organ rejection in SOT patients; not universally recommended due to adverse effects in pregnant women and limited efficiency data.	[54]
	Sofosbuvir (± Ribavirin)	Inhibits Non-structural protein 5B viral RNA polymerase; in combination with ribavirin, may partially clear the virus in chronic HEV.	Ineffective as monotherapy but shows potential in HEV genotype 3 during pregnancy; clinical value under review, and optimal dosage yet to be established.	[55]
	Zinc sulfate & zinc oxide (ZnSO₄, ZnO)	Inhibits HEV replication by targeting RNA polymerase, with virucidal activity, especially in GT1 and 3.	Demonstrated efficacy *in vitro.* Good enteric absorption and bioavailability with ZnO nanoparticles.	[56]
Investigational & Repurposed Drugs	Ifenprodil	N-methyl-D-aspartate receptor antagonist; reduces HEV RNA accumulation and viral protein expression.	Effective in liver-derived cells and in vivo models; under investigation.	[57]
	Vidofludimus calcium, Pyrazofurin	Vidofludimus calcium: dihydroorotate dehydrogenase inhibitor. Pyrazofurin: targets uridine monophosphate synthetase. Both inhibit pyrimidine synthesis enzymes, leading to disruption of HEV RNA synthesis.	Potential for treating chronic infections; Identified via drug repurposing, promising antiviral activity in preclinical settings.	[58]
	Methotrexate	An anti-inflammatory drug that inhibits HEV helicase, essential for RNA processing and replication.	Novel antiviral target for HEV; early-phase data suggest efficacy.	[59]
	NITD008 & GPC-N114	Broad-spectrum antivirals showing inhibition of HEV replication; synergistic in combination.	Promising results in vitro, particularly against genotype 1, identified via drug repurposing; further validation needed.	[60]
Management of Severe Complications	Liver Support Devices, Liver Transplantation	Provide temporary hepatic function support for severe HEV cases progressing to ALF.	Transplantation is critical when liver failure is irreversible, requires extensive postoperative care; life-saving option.	[61]

## Safeguarding tomorrow

Preventing HEV transmission in endemic regions requires enhanced sanitation, hygiene, and clean drinking water. Cooking meat above 70 °C reduces zoonotic transmission risk [20]. Fecal-oral transmission is curbed via a safe water supply, proper waste disposal, and food hygiene education [3]. Vaccine development targeting the ORF2 capsid protein has advanced, with a 56-kDa recombinant vaccine showing high efficacy in a phase II trial in Nepal, though tested in males [62]. Hecolin^®^, a 26-kDa truncated ORF2 protein vaccine produced by Xiamen Innovax Biotech Co., Ltd., in China, is the only licensed hepatitis E vaccine [63]. Approved by the China FDA in 2012, Hecolin^®^ is available for individuals over 16, especially high-risk individuals. Despite demonstrating 96% efficacy in a phase III trial, its use remains restricted to China, with the WHO calling for further phase IV studies in vulnerable populations [63]. A phase IV study in Bangladesh is evaluating Hecolin’s efficacy and safety in women of reproductive age, including pregnant women, where it has shown potential [64]. Further studies are needed to address existing concerns and facilitate the global acceptance of Hecolin^®^, alongside research on novel HEV vaccine candidates [63].

## Hurdles on the horizon: challenges in HEV vaccine development

**Fig. 5:**
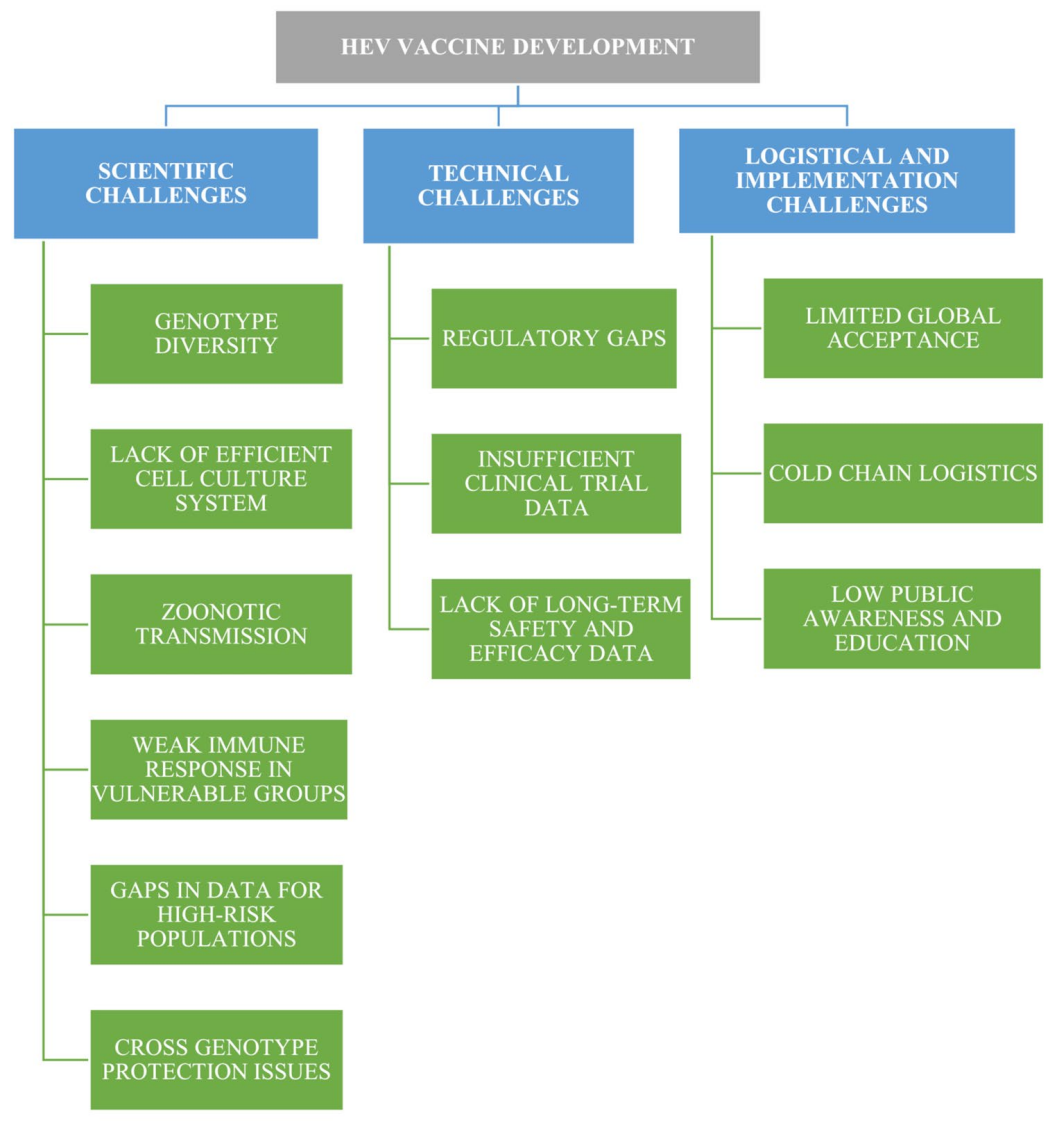
Challenges in hepatitis E vaccine development

The development of an effective hepatitis E vaccine faces key challenges due to the complex biology, diverse genotypes, and varied immune responses of the virus (Fig. 5) [65]. A major barrier is the lack of a robust cell culture system, hindering large-scale vaccine production [62, 66]. Current vaccines, such as Hecolin^®^, primarily target GT1 and offer limited protection against GT3, necessitating broader cross-genotype coverage.

The limited global availability of Hecolin^®^ underscores the need for more trials in diverse high-risk groups and solutions to logistical challenges in endemic areas. Despite two months at 30–37 °C, it still requires cold-chain storage (2–8 °C). Prefilled syringes aid dosing and reduce contamination, but complicate transport, storage, and waste management [62]. Additionally, infrastructure limitations, cost, and insufficient awareness impede vaccine integration into routine immunization schedules, especially in low- and middle-income countries [67]. Therefore, broader genotype coverage, more data in high-risk groups, and improved access are crucial for effective global HEV prevention [68].

## On trial: promising HEV vaccine candidates

The development of investigational HEV vaccines has advanced considerably, with several candidates in clinical trials. Recombinant vaccines using ORF2-based virus-like particles (VLPs) have shown promise, stimulating neutralizing antibodies [69]. Four VLP candidates have demonstrated safety and immunogenicity, particularly against GTs 1 and 4 (Table 3).

**Table 3:** List of HEV candidate vaccines and commercialized vaccines

VACCINE	MANUFACTURER	ANTIGEN	EXPRESSION SYSTEM	DOSE	EFFICACY	DEVELOPMENT STATUS	REFERENCE
(Recombinant Hepatitis E Virus) rHEV	GlaxoSmithKline	HEV-1 ORF-2 (*aa 112–607)	*Baculovirus*	0,1,6 months	>99%	Phase II	[70]
HEV 239 (Hecolin^®^)	Xiamen Innovax Biotech	HEV-1 ORF-2 (aa 368–606)	*Escherichia coli*	0,1,6 months	95.5%	Licenced Phase IV	[71, 72]
Lipo-NE-P	Zydus Lifesciences Ltd.	HEV-1 ORF-2 (aa 458–607)	*Escherichia coli*	0,1,6 months	-	Phase II	[73]
HEV P179	Changchun Institute of Biological Products Co. Ltd.	HEV-4 ORF-2 (aa 439–617)	*Escherichia coli*	0,1,6 months	-	Phase Ib	[74]

*aa- amino acid

### Combined vaccines

Dong et al. developed a combined hepatitis A and E vaccine containing inactivated HAV and recombinant HEV components. In mice, it induced neutralizing antibodies against both viruses, indicating dual protective potential [75]. Gao et al. developed a mucosal vaccine combining HAV and HEV proteins linked to tuftsin. Intranasal delivery increased IgG and IgA levels in the intestines, vagina, and lungs; increased IFN-γ levels and CD4⁺/CD8⁺ T-cell ratio, outperforming intramuscular injection. These findings support the use of HE-ORF2, HA-viral capsid protein 1, and tuftsin for improved immunogenicity in a combined hepatitis A and E vaccine [76].

### Bivalent vaccines

Li et al. developed a potential bivalent vaccine by linking an HEV capsid protein segment (aa 551–607) to hepatitis B surface antigen (HBsAg) with a synthetic glycine linker. Expressing this fusion protein in *Pichia pastoris* resulted in high immunoreactivity of the chimeric VLPs, indicating strong potential as a dual vaccine candidate against both HBV (Hepatitis B virus) and HEV [77]. Wang et al. developed a bivalent vaccine targeting norovirus (NoV) and HEV by covalently linking their dimeric P domains with dimeric glutathione-S-transferase (GST). Immunization of mice with GST-NoV P–HEV P and NoV P–HEV P complexes significantly increased antibody titres, supporting their potential as dual vaccine candidates [78]. Another study utilized a recombinant fusion protein that combines a truncated HEV ORF2 (amino acids 112–607) with rotavirus (RV) NSP4, which is expressed via a baculovirus system, suggesting another promising multivalent vaccine [79].

### Trivalent subunit vaccines

A recent study developed a trivalent subunit vaccine targeting HEV, rotavirus, and astrovirus (AstV). Two formats were tested: a “fused vaccine” combining spike protein antigens into one molecule and a “mixed vaccine” of individual antigens. Both were efficiently produced in *E. coli*. Mouse studies have shown that the fused vaccine elicits higher antibody titres and stronger neutralizing activity against HEV, alongside improved blocking of RV receptor binding. These findings highlight the use of the fused vaccine as a promising candidate for simultaneous protection against HEV, RV, and AstV [80].

### Multiple epitope peptide vaccines

Computationally designed multiepitope peptide vaccines show promise in preclinical models, inducing humoral and cellular immunity. These constructs use validated HEV epitopes and adjuvants to enhance immune responses but require further laboratory validation [81].

### Oral HEV vaccines

Recent studies have shown that oral delivery of recombinant HEV capsid proteins with immunobiotic bacterium-like particles induces strong systemic and mucosal immune responses in murine models, suggesting a needle-free HEV vaccination approach [82]. Research indicates that oral vaccines can effectively induce IgA and IgG responses, although optimal results may require combined oral and subcutaneous administration [83].

Another study evaluated the use of recombinant HEV virus-like particles (rHEV VLPs) for oral immunization. Even without a mucosal adjuvant, purified rHEV VLPs induced robust systemic (serum IgG, IgM, IgA) and mucosal (fecal IgA) responses in mice. These antibodies recognize the native HEV antigen, suggesting that rHEV VLPs are promising oral vaccine candidates [84]. Furthermore, a plant-based oral vaccine using genetically transformed tomato plants with a partial HEV ORF2 gene showed promise. Transgenic lines confirmed successful DNA integration, and the recombinant protein retained immunoactivity, highlighting transgenic tomatoes as a promising, low-cost platform for oral vaccination [85].

## Future horizons: addressing challenges and paving new paths

Hepatitis E remains a significant yet underdiagnosed public health issue in India, stemming from limited awareness and a lack of routine screening. Addressing this requires strengthened surveillance, improved diagnostics, and effective vaccination strategies. Continued research is crucial to develop safer, broadly protective vaccine candidates and address safety concerns in vulnerable groups (e.g., pregnant women and chronic liver disease patients), ensuring broader global acceptance of vaccines such as Hecolin^®^ [5].

Preventive strategies should focus on basic hygiene, safe drinking water, and proper waste disposal, alongside HEV screening in blood banks, to reduce transfusion-related transmission. A ‘One Health’ approach is essential, promoting collaboration between the human and veterinary sectors to monitor animal reservoirs such as swine, camels, and wild boar, given the diverse transmission routes of HEVs, including fecal-oral, zoonotic, parenteral, and vertical. Further research into HEV molecular virology and pathogenesis is needed to identify new therapeutic targets and develop more effective treatments for both acute and chronic infections [4, 5].

## Summing it up: insights and outlook

Hepatitis E remains a formidable and underestimated public health challenge in India. Its evolving spread, encompassing zoonotic, waterborne, and vertical routes, has placed human populations at heightened risk. The true burden often remains underestimated due to limited public awareness and a lack of standardized diagnostic methods.

To effectively curtail this disease, a multipronged approach is essential. This encompasses enhancing public awareness, facilitating early diagnosis, improving vaccination strategies, and implementing robust preventive measures. Such efforts must include strengthening surveillance systems, widespread improvements in water, sanitation, and hygiene infrastructure. Moreover, continued research into HEV pathogenesis and the development of efficacious vaccines and novel therapeutics are crucial for transforming hepatitis E control and safeguarding the public health of the nation.

## Data Availability

No datasets were generated or analysed during the current study.

## Declarations

### Generative AI and AI-assisted technologies in the writing process

During the preparation of this work, the authors used ChatGPT (https://chat.openai.com) and Gemini (https://gemini.google.com) to generate the titles for the headings of the review article. After using this tool, the authors reviewed and edited the content as needed and take full responsibility for the content of the publication.

## References

[1] Kamar N, Dalton HR, Abravanel F, Izopet J. Hepatitis E virus infection. Clin Microbiol Rev. 2014;27(1):116–38. 24396139 10.1128/CMR.00057-13 PMC3910910

[2] Dokić M, Begović V, Rajić-Dimitrijević R, Aleksić R, Popović S. Hristović D. [Fulminant hepatitis B]. Vojnosanit Pregl. 2003;60(3):353–60. 12891732 10.2298/vsp0303353d

[3] Girish V, Grant LM, Sharma B et al. Hepatitis E. [Updated 2025 Apr 6]. In: StatPearls. Treasure Island (FL): StatPearls Publishing; 2025 Jan-. Available from: https://www.ncbi.nlm.nih.gov/books/NBK532278/

[4] Yugo DM, Meng XJ, Hepatitis. E virus: foodborne, waterborne and zoonotic transmission. Int J Environ Res Public Health. 2013;10(10):4507–33. 10.3390/ijerph10104507. PMID: 24071919; PMCID: PMC3823334. 24071919 10.3390/ijerph10104507 PMC3823334

[5] Raji YE, Toung OP, Taib NM, Sekawi ZB, Hepatitis E, Virus. An emerging enigmatic and underestimated pathogen. Saudi J Biol Sci. 2022;29(1):499–512. 35002446 10.1016/j.sjbs.2021.09.003 PMC8716866

[6] Nagashima S, Takahashi M, Kobayashi T, Tanggis; Nishizawa T, Nishiyama T, Primadharsini PP, Okamoto H. Characterization of the quasi-enveloped hepatitis E virus particles released by the cellular exosomal pathway. J Virol. 2017;91(22):e00822–17. 10.1128/JVI.00822-17. PMID: 28878075; PMCID: PMC5660490.]. 28878075 10.1128/JVI.00822-17 PMC5660490

[7] Mirzaev UK, Ouoba S, Ko K, et al. Systematic review and meta-analysis of hepatitis E Seroprevalence in Southeast Asia: a comprehensive assessment of epidemiological patterns. BMC Infect Dis. 2024;24:525. 10.1186/s12879-024-09349-2 38789918 10.1186/s12879-024-09349-2 PMC11127338

[8] Noman HM, Arslan Sajid MM, Ali G, Amir MA, Akram J. Hepatitis-E outbreak in chad: a public health crisis amidst refugee influx from Sudan. World J Pharm Med Res. 2025;11(7):79–81.

[9] Sridhar S, Yip CCY, Wu S, Cai J, Zhang AJX, Leung KH, et al. Rat hepatitis E virus as cause of persistent hepatitis after liver transplant. Emerg Infect Dis. 2018;24(12):2241–50. 30457530 10.3201/eid2412.180937 PMC6256372

[10] Khuroo MS. Discovery of hepatitis E: the epidemic non-A, non-B hepatitis 30 years down the memory lane. Virus Res. 2011;161(1):3–14. 21320558 10.1016/j.virusres.2011.02.007

[11] Zhu FC, Zhang J, Zhang XF, Zhou C, Wang ZZ, Huang SJ et al. Efficacy and safety of a recombinant hepatitis E vaccine in healthy adults: a large-scale, randomized, double-blind placebo-controlled, phase 3 trial. Lancet Lond Engl. 2010;376(9744):895–902. 10.1016/S0140-6736(10)61030-6 20728932

[12] Koyuncu A, Mapemba D, Ciglenecki I, Gurley ES, Azman AS. Setting a course for preventing hepatitis E in low and lower-middle-income countries: a systematic review of burden and risk factors. InOpen Forum Infectious Diseases. 2021;8(6):ofab178. US: Oxford University Press. 10.1093/ofid/ofab178 PMC8186248 34113684

[13] Teshale EH, Hu DJ, Hepatitis E. Epidemiology and prevention. World J Hepatol. 2011;3(12):285–91. 10.4254/wjh.v3.i12.285. PMID: 22216368; PMCID: PMC3246546. 22216368 10.4254/wjh.v3.i12.285 PMC3246546

[14] Kadri SM, Rehman SU, Rehana K, Benetou DR, Ahmad DF, Abdullah A, Chattu VK. Hepatitis A and E outbreak surveillance during 2015–2017 in Kashmir, india: is the water to blame? J Epidemiol Global Health. 2018;8(3):204–7. 10.2991/j.jegh.2018.04.101 PMC7377564 30864764

[15] Nan Y, Wu C, Zhao Q, Zhou EM. Zoonotic hepatitis E virus: an ignored risk for public health. Front Microbiol. 2017;8:2396. 29255453 10.3389/fmicb.2017.02396 PMC5723051

[16] Kamani L, Padhani ZA, Das JK, Hepatitis E. Genotypes, strategies to prevent and manage, and the existing knowledge gaps. JGH Open Open Access J Gastroenterol Hepatol. 2021;5(10):1127–34. 10.1002/jgh3.12646 PMC8485408 34621997

[17] Virhuez-Mendoza M, Ishijima K, Tatemoto K et al. Recent hepatitis E virus infection in wild boars and other ungulates in Japan. Viruses. 2025;17(4):524. 10.3390/v17040524. PMID: 40284967. 10.3390/v17040524 PMC12031028 40284967

[18] Santos-Silva S, Hemnani M, Lopez-Lopez P et al. A systematic review of hepatitis E virus detection in camels. Veterinary Sciences. 2023;10(5):323. 10.3390/vetsci10050323. PMID: 37235406. 10.3390/vetsci10050323 PMC10222403 37235406

[19] Zhang W, Li S, Shu X et al. A cross-species transmission of a camel-derived genotype 8 hepatitis E virus to rabbits. Pathogens. 2021;10(11):1374. 10.3390/pathogens10111374. PMID: 34832530. 10.3390/pathogens10111374 PMC8618709 34832530

[20] Naik SR, Aggarwal R, Salunke PN, Mehrotra NN. A large waterborne viral hepatitis E epidemic in Kanpur, India. Bull World Health Organ. 1992;70(5):597–604. PMID: 1464145; PMCID: PMC2393368. 1464145 PMC2393368

[21] Kumar T, Shrivastava A, Kumar A, Laserson KF, Narain JP, Venkatesh S, Chauhan LS, Averhoff F. Viral hepatitis surveillance–India, 2011–2013. MMWR Morb Mortal Wkly Rep. 2015;64(28):758–62. 10.15585/mmwr.mm6428a3. PMID: 26203629; PMCID: PMC4584861. 26203629 10.15585/mmwr.mm6428a3 PMC4584861

[22] Negi SS, Barde PV, Pathak R, Gaikwad U, Das P, Bhargav A. An outbreak of hepatitis E virus in Raipur, Chhattisgarh, India in 2014: a conventional and genetic analysis. J Med Microb Diagn. 2015;4(209):2161–0703.

[23] Daniel R, Zelber-Sagi S, Barak M, Zuckerman E. The epidemiology of hepatitis E in Israel and potential risk factors: a cross-sectional population-based serological survey of hepatitis E virus in Northern Israel. Viruses. 2025;17(4):536. 40284979 10.3390/v17040536 PMC12031424

[24] Wu C, Wu X, Xia J. Hepatitis E virus infection during pregnancy. Virol J. 2020;17:73. 10.1186/s12985-020-01343-9 32522266 10.1186/s12985-020-01343-9 PMC7286216

[25] Raji YE, Toung OP, Mohd Taib N, Sekawi ZB. A systematic review of the epidemiology of hepatitis E virus infection in South - Eastern Asia. Virulence. 2021;12(1):114–29. PMID: 33372843; PMCID: PMC7781573. 33372843 10.1080/21505594.2020.1865716 PMC7781573

[26] Nasir M, Wu GY. HEV and HBV dual infection: a review. J Clin Transl Hepatol. 2020;8(3):313–321. 10.14218/JCTH.2020.00030. Epub 2020 Jul 3. PMID: 33083255; PMCID: PMC7562801. 10.14218/JCTH.2020.00030 PMC7562801 33083255

[27] Takuissu GR, Kenmoe S, Ndip L, Ebogo-Belobo JT, Kengne-Ndé C, Mbaga DS, et al. Hepatitis E virus in water environments: a systematic review and meta-analysis. Food Environ Virol. 2022;14(3):223–35. 36036329 10.1007/s12560-022-09530-3 PMC9458591

[28] Treagus S, Wright C, Baker-Austin C, Longdon B, Lowther J. The foodborne transmission of hepatitis E virus to humans. Food Environ Virol. 2021;13(1):127–45. 10.1007/s12560-021-09461-5 PMC8116281 33738770

[29] Webb GW, Dalton HR. Hepatitis E: an expanding epidemic with a range of complications. Clin Microbiol Infect. 2020;26(7):828–32. 10.1016/j.cmi.2020.03.039 32251845

[30] Aslan AT, Balaban HY. Hepatitis E virus: epidemiology, diagnosis, clinical manifestations, and treatment. World J Gastroenterol. 2020;26(37):5543–60. 33071523 10.3748/wjg.v26.i37.5543 PMC7545399

[31] Xin S, Xiao L. Clinical Manifestations of Hepatitis E. In: Wang Y, editor. Hepatitis E virus. Dordrecht: Springer Netherlands; 2016 [cited 2025 May 28]. pp. 175–89. Available from: 10.1007/978-94-024-0942-0_10 10.1007/978-94-024-0942-0_10 27738985

[32] Bernal W, Wendon J. Acute liver failure. N Engl J Med. 2013;369(26):2525–34. 24369077 10.1056/NEJMra1208937

[33] Murali AR, Kotwal V, Chawla S. Chronic hepatitis E: a brief review. World J Hepatol. 2015;7(19):2194–201. 10.4254/wjh.v7.i19.2194 PMC4561773 26380044

[34] Chaudhry SA, Verma N, Koren G. Hepatitis E infection during pregnancy. Can Fam Physician. 2015;61(7):607–8. PMID: 26175368; PMCID: PMC4501603. 26175368 PMC4501603

[35] Taneja S, Sen S, Gupta VK, Aggarwal R, Jameel S. Plasma and urine biomarkers in acute viral hepatitis E. Proteome Sci. 2009;7(1):39. 19860894 10.1186/1477-5956-7-39 PMC2773234

[36] Costafreda MI, Sauleda S, Riveiro-Barciela M, Rico A, Llorens-Revull M, Guix S, Pintó RM, Bosch A, Rodríguez-Frías F, Rando A, Piron M, Bes M. Specific plasma MicroRNA signatures underlying the clinical outcomes of hepatitis E virus infection. Microbiol Spectr. 2023;11(1):e0466422. 10.1128/spectrum.04664-22. Epub 2023 Jan 25. PMID: 36695578; PMCID: PMC9927377. 36695578 10.1128/spectrum.04664-22 PMC9927377

[37] Huang S, Zhang X, Jiang H, Yan Q, Ai X, Wang Y, Cai J, Jiang L, Wu T, Wang Z, Guan L, Kuo Shih JW, Ng H, Zhu F, Zhang J, Xia N. Profile of acute infectious markers in sporadic hepatitis E. PLoS ONE. 2010;5(10):e13560. 10.1371/journal.pone.0013560 21042408 10.1371/journal.pone.0013560 PMC2958841

[38] Copado-Villagrana ED, Pizuorno A, García-Suárez A, Abarca JC, DuPont G, Jaramillo-Bueno S et al. IL-18 discriminates highly frequent hepatitis E virus positive from negative blood donors in Mexico. Ann Hepatol. 2023. [cited 2025 May 27];28(5). Available from: http://www.elsevier.es/en-revista-annals-hepatology-16-articulo-il-18-discriminates-highly-frequent-hepatitis-S1665268123002211 10.1016/j.aohep.2023.101117 37268060

[39] EASL Clinical Practice Guidelines on hepatitis E virus infection. J Hepatol. 2018;68(6):1256–71. 10.1016/j.jhep.2018.03.005 29609832 10.1016/j.jhep.2018.03.005

[40] Takakusagi S, Kakizaki S, Takagi H. The diagnosis, pathophysiology, and treatment of chronic hepatitis E virus infection—a condition affecting immunocompromised patients. Microorganisms. 2023;11(5):1303. 37317277 10.3390/microorganisms11051303 PMC10220693

[41] Iqbal H, Mehmood BF, Sohal A, Roytman M. Hepatitis E infection: a review. World J Virol. 2023;12(5):262. 38187497 10.5501/wjv.v12.i5.262 PMC10768387

[42] National Institute of Diabetes and Digestive and Kidney Diseases (US). LiverTox: clinical and research information on drug-induced liver injury. National Institute of Diabetes and Digestive and Kidney Diseases; 2012. 31643176

[43] WHO Guidelines on GMP for Blood Establishments. Annex 4 (2023). https://www.who.int/docs/default-source/medicines/norms-and-standards/guidelines/production/trs961-annex4-gmp-blood-establishments.pdf

[44] A method for developing a rapid immunochromatographic assay for identifying hepatitis E infection (ICMR-NIV HEV IgM Rapid Test). (Diagnostic Assay/Kit). https://www.icmr.gov.in/icmrobject/custom_data/1711646578_eoi05_mdms_extended.pdf

[45] Fan Z, Xu L, Cao Y, Liu T, Tian Y, Pan Z, Mo Y, Wang X, Zhu X, Gao Y, Zhang X, Pan CQ, Wang L, Ren F. One-pot assay based on CRISPR/Cas13a technology for HEV RNA point-of-care testing. J Med Virol. 2024;96(12):e70115. 10.1002/jmv.70115. PMID: 39704190; PMCID: PMC11660031. 39704190 10.1002/jmv.70115 PMC11660031

[46] Development of a point of care lateral flow diagnostic to test for acute and past hepatitis E (Hev) In Saliva | National Agricultural Library. [cited 2025 Apr 9]. Available from: https://www.nal.usda.gov/research-tools/food-safety-research-projects/development-point-care-lateral-flow-diagnostic-test

[47] Ying D, Hong C, Wen G, Tang Z, Wang S, Zhang X, et al. Development and evaluation of a rapid point-of-care test for detecting the hepatitis E virus antigen. Clin Biochem. 2018;55:89–92. 29518382 10.1016/j.clinbiochem.2018.03.001

[48] Li M, Li T, Hao X, Liu Y, Lan H, Zhou C. Preliminary investigation of hepatitis E virus detection by a recombinase polymerase amplification assay combined with a lateral flow strip. J Veterinary Diagn Investigation: Official Publication Am Association Veterinary Lab Diagnosticians Inc. 2023;35(4):395–8. 10.1177/10406387231167119 10.1177/10406387231167119 PMC10331385 37029661

[49] Hao X, Liu Y, Lan H, Zhou C. Preliminary investigation of hepatitis E virus detection by a recombinase polymerase amplification assay combined with a lateral flow strip. J Veterinary Diagn Investigation: Official Publication Am Association Veterinary Lab Diagnosticians Inc. 2023;35(4):395–8. 10.1177/10406387231167119 10.1177/10406387231167119 PMC10331385 37029661

[50] Li M, He Q, Li T, Wan W, Zhou H. Development and evaluation of a CRISPR-Cas13a system-based diagnostic for hepatitis E virus. Clin Chem Lab Med. 2023;62(6):1237–1247. 10.1515/cclm-2023-1007. PMID: 38153113. 10.1515/cclm-2023-1007 38153113

[51] Wei B, Wang W, Guo Z, Yin W, Cheng M, Yang Y, Tian Y, Sun Y, Liu T, Hu Y, She R. Rapid visual detection of hepatitis E virus combining reverse transcription recombinase-aided amplification with lateral flow dipstick and real-time fluorescence. J Clin Microbiol. 2025;63(2):e01064–24. 39817756 10.1128/jcm.01064-24 PMC11837526

[52] Hui W, Wei L, Li Z, Guo X. Treatment of hepatitis E. Hepat E Virus. 2016;14:211–21. 10.1007/978-94-024-0942-0_12 27738987

[53] Kamar N, Izopet J, Tripon S, et al. Ribavirin for chronic hepatitis E virus infection in transplant recipients. N Engl J Med. 2014;370(12):1111–20. 24645943 10.1056/NEJMoa1215246

[54] Ollivier-Hourmand I, Lebedel L, Lecouf A, Allaire M, Nguyen TTN, Lier C, Dao T. Pegylated interferon may be considered in chronic viral hepatitis E resistant to ribavirin in kidney transplant recipients. BMC Infect Dis. 2020;20(1):522. 10.1186/s12879-020-05212-2. PMID: 32677900; PMCID: PMC7367388. 32677900 10.1186/s12879-020-05212-2 PMC7367388

[55] Lampejo T. Sofosbuvir in the treatment of hepatitis E virus infection: a review of in vitro and in vivo evidence. J Clin Exp Hepatol. 2022;12(4):1225–37. 10.1016/j.jceh.2022.02.003. Epub 2022 Feb 22. PMID: 35814503; PMCID: PMC9257862. 10.1016/j.jceh.2022.02.003 PMC9257862 35814503

[56] Kaushik N, Subramani C, Anang S, Muthumohan R, Shalimar, Nayak B, Ranjith-Kumar CT, Surjit M. Zinc salts block hepatitis E virus replication by inhibiting the activity of viral RNA-dependent RNA polymerase. J Virol. 2017;91(21):e00754–17. 10.1128/JVI.00754-17. PMID: 28814517; PMCID: PMC5640865. 28814517 10.1128/JVI.00754-17 PMC5640865

[57] Klöhn M, Gömer A, He Q, Brown RJP, Todt D, Wang L, Steinmann E. The glutamate receptor antagonist ifenprodil inhibits hepatitis E virus infection. Antimicrob Agents Chemother. 2024;68(11):e0103524. 10.1128/aac.01035-24. Epub 2024 Oct 3. PMID: 39360823; PMCID: PMC11539220. 39360823 10.1128/aac.01035-24 PMC11539220

[58] Guo H, Liu D, Liu K, Hou Y, Li C, Li Q, Ding X, Verstegen MMA, Zhang J, Wang L, Ding Y, Tang R, Pan X, Zheng K, van der Laan LJW, Pan Q, Wang W. Drug repurposing screen identifies vidofludimus calcium and pyrazofurin as novel chemical entities for the development of hepatitis E interventions. Virol Sin. 2024;39(1):123–33. Epub 2023 Nov 19. PMID: 37984761; PMCID: PMC10877426. 37984761 10.1016/j.virs.2023.11.006 PMC10877426

[59] Kumar A, Hooda P, Puri A, Khatter R, Al-Dosari S, Sinha M, Parvez N, Sehgal MK. Methotrexate, an anti-inflammatory drug, inhibits hepatitis E viral replication. J Enzyme Inhib Med Chem. 2023;38(1):2280500. Epub 2023 Nov 17. PMID: 37975328; PMCID: PMC11003484. 37975328 10.1080/14756366.2023.2280500 PMC11003484

[60] Netzler NE, Enosi Tuipulotu D, Vasudevan SG, Mackenzie JM, White PA. Antiviral candidates for treating hepatitis E virus infection. Antimicrob Agents Chemother. 2019;63(6):e00003–19. 10.1128/AAC.00003-19. PMID: 30885901; PMCID: PMC6535575. 30885901 10.1128/AAC.00003-19 PMC6535575

[61] Larsen FS, Saliba F. Liver support systems and liver transplantation in acute liver failure. Liver Int. 2025;45(3):e15633. 10.1111/liv.15633. Epub 2023 Jun 8. PMID: 37288706; PMCID: PMC11815598. 37288706 10.1111/liv.15633 PMC11815598

[62] Huang X, Lu J, Liao M, Huang Y, Wu T, Xia N. Progress and challenges to hepatitis E vaccine development and deployment. Vaccines. 2024;12(7):719. 10.3390/vaccines12070719 PMC11281425 39066357

[63] Wu X, Chen P, Lin H, Hao X, Liang Z. Hepatitis E virus: current epidemiology and vaccine. Hum Vaccin Immunother. 2016;12(10):2603–10. 10.1080/21645515.2016.1184806. Epub 2016 May 16. PMID: 27184971; PMCID: PMC5085000. 27184971 10.1080/21645515.2016.1184806 PMC5085000

[64] Zaman K, Dudman S, Stene-Johansen K, Qadri F, Yunus M, Sandbu S, Gurley ES, Overbo J, Julin CH, Dembinski JL, Nahar Q. HEV study protocol: design of a cluster-randomised, blinded trial to assess the safety, immunogenicity and effectiveness of the hepatitis E vaccine HEV 239 (Hecolin) in women of childbearing age in rural Bangladesh. BMJ Open. 2020;10(1):e033702. 31959609 10.1136/bmjopen-2019-033702 PMC7044974

[65] Behrendt P, Wedemeyer H. Vaccines against hepatitis E virus: state of development. Bundesgesundheitsblatt Gesundheitsforschung Gesundheitsschutz. 2022;65(2):192–201. 35099576 10.1007/s00103-022-03487-1 PMC8802100

[66] Ahmad T, Haroon H, Ahmad K, Shah SM, Shah MW, Hussain A et al. Hepatitis E vaccines: a mini review. Biomed Res Ther. 2021;8(9):4514–24.

[67] Dudman S, Zerja A, Hasanoğlu İ, Ruta S, van Welzen B, Nicolini LA, et al. Global vaccination against hepatitis E virus: position paper from the European Society of Clinical Microbiology and Infectious Diseases Viral Hepatitis Study Group. Clin Microbiol Infect. 2025;31(2):201–10. 39550032 10.1016/j.cmi.2024.11.016

[68] Kamili S. Toward the development of a hepatitis E vaccine. Virus Res. 2011;161(1):93–100. 21620908 10.1016/j.virusres.2011.05.008

[69] Mazalovska M, Kouokam JC. Progress in the production of virus-like particles for vaccination against hepatitis E virus. Viruses. 2020;12(8):826. 10.3390/v12080826. PMID: 32751441; PMCID: PMC7472025. 32751441 10.3390/v12080826 PMC7472025

[70] Shrestha MP, Scott RM, Joshi DM, Mammen MP, Thapa GB, Thapa N, et al. Safety and efficacy of a recombinant hepatitis E vaccine. N Engl J Med. 2007;356(9):895–903. 17329696 10.1056/NEJMoa061847

[71] Zhang J, Zhang XF, Huang SJ, Wu T, Hu YM, Wang ZZ, et al. Long-term efficacy of a hepatitis E vaccine. N Engl J Med. 2015;372(10):914–22. 25738667 10.1056/NEJMoa1406011

[72] Huang S, Zhang X, Su Y, Zhuang C, Tang Z, Huang X, et al. Long-term efficacy of a recombinant hepatitis E vaccine in adults: 10-year results from a randomized, double-blind, placebo-controlled, phase 3 trial. Lancet Lond Engl. 2024;403(10429):813–23. 10.1016/S0140-6736(23)02234-1 38387470

[73] CTRI. [cited 2025 June 5]. Available from: https://www.ctri.nic.in/Clinicaltrials/pmaindet2.php?EncHid=ODAwMjg=%26;Enc=%26;userName=Adsorbed

[74] Cao YF, Tao H, Hu YM, Shi CB, Wu X, Liang Q et al. A phase 1 randomized open-label clinical study to evaluate the safety and tolerability of a novel Recombinant hepatitis E vaccine. Vaccine. 2017;35(37):5073–80. 10.1016/j.vaccine.2017.05.072 28803715

[75] Dong C, Dai X, Meng JH. The first experimental study on a candidate combined vaccine against hepatitis A and hepatitis E. Vaccine. 2007;25(9):1662–8. 17156900 10.1016/j.vaccine.2006.11.001

[76] Gao Y, Su Q, Yi Y, Jia Z, Wang H, Lu X, et al. Enhanced mucosal immune responses induced by a combined candidate mucosal vaccine based on hepatitis A virus and hepatitis E virus structural proteins linked to Tuftsin. PLoS ONE. 2015;10(4):e0123400. 25875115 10.1371/journal.pone.0123400 PMC4395237

[77] Li HZ, Gang HY, Sun QM, Liu X, Ma YB, Sun MS, Dai CB. Production in Pichia pastoris and characterization of genetic engineered chimeric HBV/HEV virus-like particles. Chin Med Sci J. 2004;19(2):78–83. PMID: 15250239. 15250239

[78] Wang L, Cao D, Wei C, Meng XJ, Jiang X, Tan M. A dual vaccine candidate against norovirus and hepatitis E virus. Vaccine. 2014;32(4):445–52. 10.1016/j.vaccine.2013.11.064. Epub 2013 Nov 27. PMID: 24291540; PMCID: PMC3898346. 24291540 10.1016/j.vaccine.2013.11.064 PMC3898346

[79] Makvandi M, Teimoori A, Neisi N, Samarbafzadeh A, Designing. Construction and expression of a recombinant fusion protein comprising the hepatitis E virus ORF2 and rotavirus NSP4 in the baculovirus expression system. Jundishapur J Microbiol. 2016;9(11):e40303. 10.5812/jjm.40303. PMID: 28138375; PMCID: PMC5240165. 28138375 10.5812/jjm.40303 PMC5240165

[80] Xia M, Wei C, Wang L, Cao D, Meng XJ, Jiang X, et al. Development and evaluation of two subunit vaccine candidates containing antigens of hepatitis E virus, rotavirus, and astrovirus. Sci Rep. 2016;6:25735. 27194006 10.1038/srep25735 PMC4872161

[81] Anwar T, Ismail S, Parvaiz F, Abbasi SW, Al-Abbasi FA, Alghamdi AM, et al. Computational design of experimentally validated multiepitopes vaccine against hepatitis E virus: an immunological approach. PLoS ONE. 2023;18(12):e0294663. 38096182 10.1371/journal.pone.0294663 PMC10721065

[82] Arce LP, Raya Tonetti MF, Raimondo MP, Müller MF, Salva S, Álvarez S, Baiker A, Villena J, Vizoso Pinto MG. Oral vaccination with hepatitis E virus capsid protein and immunobiotic bacterium-like particles induce intestinal and systemic immunity in mice. Probiotics Antimicrob Proteins. 2020;12(3):961–72. 31630331 10.1007/s12602-019-09598-7

[83] Müller MF, Sacur J, Brancher JM, Vera MD, Arce L, Raya-Tonetti MF, et al. An experimental chimeric hepatitis E virus vaccine elicits both local and systemic immune responses. Front Microbiol. 2024;15:1512018. 39777142 10.3389/fmicb.2024.1512018 PMC11704494

[84] Li T, Takeda N, Miyamura T. Oral administration of hepatitis E virus-like particles induces a systemic and mucosal immune response in mice. Vaccine. 2001;19(25–26):3476-84. 10.1016/s0264-410x(01)00059-7. PMID: 11348714. 10.1016/s0264-410x(01)00059-7 11348714

[85] Ma Y, Lin SQ, Gao Y, Li M, Luo WX, Zhang J, Xia NS. Expression of ORF2 partial gene of hepatitis E virus in tomatoes and immunoactivity of expression products. World J Gastroenterol. 2003;9(10):2211–5. 10.3748/wjg.v9.i10.2211 14562380 10.3748/wjg.v9.i10.2211 PMC4656465

